# Therapies with Emerging Evidence of Efficacy: Avotermin for the Improvement of Scarring

**DOI:** 10.1155/2010/690613

**Published:** 2010-08-03

**Authors:** Jim Bush, Karen So, Tracey Mason, Nick L. Occleston, Sharon O'Kane, Mark W. J. Ferguson

**Affiliations:** Renovo, Core Technology Facility, 48 Grafton Street, Manchester M13 9XX, UK

## Abstract

Many patients are dissatisfied with scars on both visible and non-visible body sites and would value any opportunity to improve or minimise scarring following surgery. Approximately 44 million procedures in the US and 42 million procedures in the EU per annum could benefit from scar reduction therapy. A wide range of non-invasive and invasive techniques have been used in an attempt to improve scarring although robust, prospective clinical trials to support the efficacy of these therapies are lacking. Differences in wound healing and scar outcome between early fetal and adult wounds led to interest in the role of the TGF*β* family of cytokines in scar formation and the identification of TGF*β*3 (avotermin) as a potential therapeutic agent for the improvement of scar appearance. Extensive pre-clinical and human Phase I and II clinical trial programmes have confirmed the scar improving efficacy of avotermin which produces macroscopic and histological improvements in scar architecture, with improved restitution of the epidermis and an organisation of dermal extracellular matrix that more closely resembles normal skin. Avotermin is safe and well tolerated and is currently in Phase III of clinical development, with the first study, in patients undergoing scar revision surgery, fully recruited.

## 1. There Is a Medical Need for Therapies That Reduce Scarring following Surgery

A recent survey performed in the USA confirmed that many patients are disappointed with their scar resulting from a surgical procedure, irrespective of gender, age or ethnicity [1]. Understandably, patients are very conscious about visible scars. However, the survey showed that it is not only visible scars that cause dissatisfaction, with many patients reporting scars on nonvisible body sites that they wished were less noticeable [1]. A patient's perception of the severity of their scar can be influenced not only by the objective appearance of the scar, but also by other factors including the surgical technique used and the patient's sensitivity to the resulting scar [1, 2]. Many patients would value any opportunity to improve or minimise scarring following surgery [1]. Indeed it was estimated that there are approximately 44 million procedures performed in the US (Independent research: Mattson Jack Group) and approximately 42 million procedures performed in the EU per annum (Independent research for Renovo: MedTech Insights and TforG) that could benefit from scar reduction therapy. 

## 2. Current Treatments for Scar Management Are Unsatisfactory

The optimal outcome of wound repair following trauma, injury or surgery is complete restoration of normal skin. However, adult wound healing has evolved to rapidly replace missing tissue with repaired tissue, consisting predominantly of fibronectin and collagen types I and III. The repaired tissue provides an immediate barrier to foreign bodies and infectious agents, irrespective of optimal function or appearance [3]. However, in the context of modern surgery, which is performed under sterile conditions, this immediate barrier is unnecessary and therefore scarring can be considered an inappropriate response. Furthermore, dermal scarring can have significant adverse consequences including restriction of movement and psychological trauma. 

A number of different approaches have been used in an effort to manage scarring post surgery ranging from noninvasive (silicone gel sheeting, pressure garments, hydrating creams, and ointments) to invasive (steroid injections, lasers, dermabrasion, and surgery) techniques [4–9] (Table 1). Unfortunately, many of these approaches are uncomfortable or burdensome for the patients, and many require a high level of patient compliance. Furthermore, prospective, robust clinical trials to demonstrate the efficacy of scar therapies are lacking, with the majority of published studies providing only level 4 evidence [10]. Without clear definitions of criteria for scar improvement, coupled with the heterogenous nature of scars themselves, it is difficult to interpret and compare data from these studies. Consequently, no single treatment or regimen has been universally adopted as the standard of care to manage scarring post surgery. The high level of dissatisfaction with scar therapies among patients is reflected by the high number of patients who undergo scar revision surgery, estimated to be over 150,000 per annum in the USA (http://www.yourplasticsurgeryguide.com/trends/asps-2007.htm). Indeed many more patients are believed to request scar revision surgery but are refused as the clinician believes improvement is unlikely with surgery alone. 

## 3. New Biological Approaches Are in Development for the Prophylactic Improvement of Scarring

An increase in our understanding of the processes involved in scarring at the molecular, cellular, and tissue levels has facilitated the development of new pharmaceutical approaches to prevent or treat scarring. While the majority of these approaches are still being investigated in the laboratory, a few have progressed to human clinical trials (Table 2) [9]. To date there is no approved pharmaceutical product in the US or the EU indicated for the reduction, improvement, or prevention of dermal scarring. 

### 3.1. The TGF*β* Isoforms Play a Key Role in Scar Formation

Scarring in adult skin is a macroscopic disturbance of the normal structure and function of the skin architecture that results from a healed wound [11], although the severity of the resulting scars differs between people and body locations. By contrast, skin wounds on early mammalian embryos have been shown to heal perfectly with no signs of scarring. The transition between scar-free healing (e.g., in early embryonic wounds), to scar-forming healing, (e.g., in adults) is characterised by a change in the organisation of the dermal extracellular matrix from a normal basket weave orientation to the deposition of parallel bundles of fine collagen fibres that form a scar [11].

There are a large number of differences between early fetal and adult wounds, the majority of which appear to be irrelevant to healing and scar formation. However, much effort has gone into identifying factors that play a causative role in the scar-free healing phenotype [12]. 

In particular, the Transforming Growth Factor beta (TGF*β*) isoforms have been shown to play a key role in determining the scarring outcome. The high ratio of TGF*β*3 to TGF*β*1 and *β*2 in embryonic wounds that heal without a scar compared with adult wounds that scar [13], led to interest in the role of this family of cytokines in scar formation. More recently, a significantly higher ratio of TGF*β*3 to the other TGF*β* isoforms has been shown in the adult oral mucosa, also known to heal with minimal scarring, compared with dermal wounds [14]. Conversely, TGF*β*1 and *β*2 are elevated in adult wounds that heal with a scar compared with embryonic wounds that heal without a scar [15]. The addition of TGF*β*1 to a rat fetal wound that would normally heal without a scar results in scar formation [16], whereas in adult rats, scarring can be reduced by the inhibition of TGF*β*1 or TGF*β*2 using antibodies or the addition of TGF*β*3[17, 18]. Furthermore, mice embryos that are genetically null for TGF*β*3 heal with a scar in comparison with wild-type littermates (with two normal copies of the TGF*β*3 genes), which exhibit scar-free healing [19].

**Figure 1:**
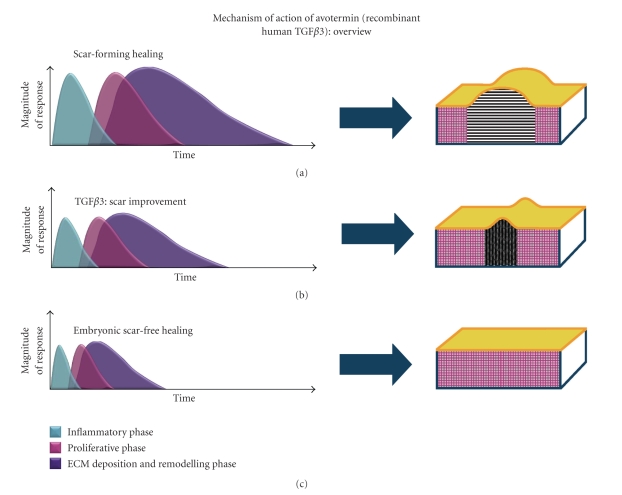
Effect of TGF*β*3 on the duration and magnitude of the scar-forming healing response. There are typically three overlapping phases involved in healing and scarring: inflammatory phase (blue), proliferative phase (pink), and deposition and remodelling phase (purple). TGF*β*3 reduces both the magnitude and the duration of each of the phases, resulting in a permanent change in tissue architecture such that collagen within the dermis is arranged in a more “basket weave” orientation (b), unlike the closely packed, parallel bundles of collagen that are characteristic of adult scar-forming healing (a). Consequently, the application of TGF*β*3 to adult wounds results in a phenotype that more closely resembles that of normal skin (c).

Collectively, data from these experimental manipulations suggest that TGF*β*3 plays an important role in scar-free healing and that the application of this cytokine to adult wounds may reduce the magnitude and accelerate the resolution of the scarring response, resulting in a phenotype that more closely resembles that of normal skin [3] (Figure 1).

### 3.2. Preclinical Studies Have Demonstrated the Efficacy and Safety of Avotermin (TGF*β*3) for the Improvement of Scar Appearance

An extensive, preclinical programme has investigated the feasibility of therapeutically manipulating the scarring response using human recombinant TGF*β*3, avotermin (Juvista^®^: Renovo, UK) to improve the appearance of scars [20]. This preclinical programme was facilitated by the high level of amino acid homology between humans and animals for TGF*β*3 and the surface receptors through which it exerts its biological effects (TGF*β* receptors type I and type II). A standardised rat model was used to investigate the efficacy of avotermin for the improvement of scarring. Intradermal injection of avotermin (50 and 100 ng/100 *μ*L/linear cm) to cutaneous incisional wounds significantly reduced scarring compared with controls in adult rats [18]. The macroscopic improvements in scarring achieved with avotermin were accompanied by histological improvements in the architecture of the neodermis, including a more normal basket weave arrangement of collagen [2, 18, 19]. The rat model was also used to optimise the dose, frequency, and formulation of avotermin, demonstrating that two injections of avotermin (50 and 100 ng/100 *μ*L/linear cm) administered at the time of wounding and 24 hours later, resulted in the greatest improvements in scarring compared with controls. A comprehensive preclinical safety program to support the development of avotermin was also completed. Specific safety studies in a clinically relevant pig model also demonstrated that intradermal administration of avotermin, at concentrations >12 times higher than those shown to be efficacious in people, is well tolerated, does not adversely affect wound healing or wound tensile strength, has low systemic bioavailability and is rapidly cleared with no systemic toxicity [21]. 

### 3.3. Avotermin Is the First in a New Class of Prophylactic Medicines in Clinical Development to Improve Scar Appearance

Following encouraging efficacy and safety data from the preclinical studies, an extensive phase I/II clinical trial programme was executed to evaluate the safety and optimise both the administration of avotermin and the design of clinical studies to assess its scar improving effects. 

The phase I/II trial programme included a series of prospective, double-blind, within-subject placebo-controlled, randomised clinical trials in human volunteers and patients. A number of parameters that can influence the appearance of scars (e.g. age, race, sex, anatomical location, etc.) have been identified. To overcome scar variability due to these parameters, a within-subject trial design, which allowed the effect of the drug to be compared with placebo across anatomically matched pairs of scars, was used in the phase I/II clinical programme. This within-subject design controlled for genetic and environmental factors affecting wound healing and scarring between individuals. By exploring a number of ways of assessing scars that result from experimental wounds, robust endpoints for the assessment of scarring have been developed and validated. In addition to assessing standard, objective endpoints for example, scar redness, pigmentation, width, height, volume, surface area [22, 23], more holistic assessments of scars have been developed. A visual analogue scale was used along with scar ranking to assess scars either on the patients (assessed by either the investigator or the patient themselves) or using standardised digital images (assessed by panels of clinicians/lay people). The visual analogue scale and scar ranking assessments have been shown to be robust and sensitive methods for accurately assessing dermal scarring [24]. During the phase I/II programme, an improved scar assessment tool was developed, the Global Scar Comparison Scale (GSCS) specifically for use in within-subject controlled trials. The GSCS incorporates the well-established principles of a visual analogue scale, with the benefits of scar ranking to provide a more accurate and sensitive measure of treatment effect with clinical relevance. The scale allows assessors to indicate which of two scars on the same subject is better and by how much, and is effectively a double VAS scale, symmetrical around a zero point, with one end of the scale used to indicate that one scar is better and the other end of the scale used to indicate that the other scar is better. The midpoint of the scale is used to indicate that there is no difference between the two scars and scores further away from the midpoint indicate a greater difference between two scars. The GSCS is also a holistic assessment tool; assessors are asked to take all scar features into consideration including scar width, height, contour, and colour. Both the within-patient design and use of the GSCS, which has now been validated in a number of phase II studies, received favourable feedback from the European Medicines Agency (EMEA) and will be used to assess avotermin further in phase III studies. 

In all clinical studies, avotermin was administered around the time of surgery as an intradermal injection either along the planned line of incision or down both margins of a closed wound, to direct delivery and ensure accurate dosing. The data collected from more than 1,100 subjects who have been exposed to avotermin during the extensive phase I/II programme demonstrate that avotermin is well tolerated with a favourable safety profile. Furthermore, no adverse effects on normal healing have been reported. To date, seven double-blind, placebo-controlled, prospective trials have met their primary endpoint, demonstrating a statistically significant improvement in scarring with avotermin. (These trials are registered with ClinicalTrials.gov: NCT00847925, NCT00847795, NCT00432211, NCT00629811, NCT00627536, NCT00594581, and NCT00430326.)

**Figure 2:**
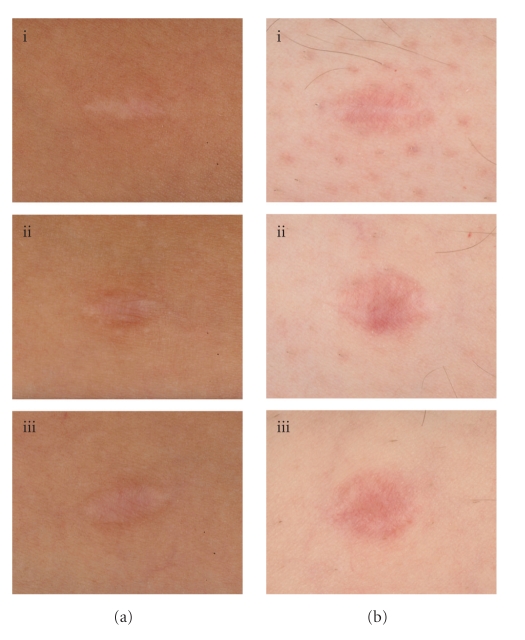
Photographic images showing the improvement in scar appearance with avotermin versus placebo and standard care. Two patients (one with paler skin (b)) with wounds treated with avotermin 200 ng/100 *μ*l/linear cm of wound margin immediately before surgery and 24 hours later (i), placebo (ii), and standard care (iii) at Month 12.

Intradermal avotermin has a broad efficacious dose range with doses of 50 to 500 ng/100*μ*L per linear cm of wound margin, administered around the time of surgery, significantly improving scar appearance [25] (Figure 2). Although effective following a single application, optimal efficacy is achieved using a twice dosing regimen, the first dose given at the time of wounding and the second dose 24 hours later. The rationale for dosing at the time of wounding is to influence the initial cascade of molecular and cellular processes involved in wound healing and scarring that are triggered immediately after wounding. A subsequent administration, 24 hours later, has been shown to provide further improvements in scarring compared with placebo, which are apparent after only 6 weeks and are maintained beyond one year [25]. Dosing schedules involving more than two administrations have been assessed and found to be sub-optimal due to potential injury caused by repeated injections at the wound site and inconvenient due to multiple clinic visits.

**Figure 3:**
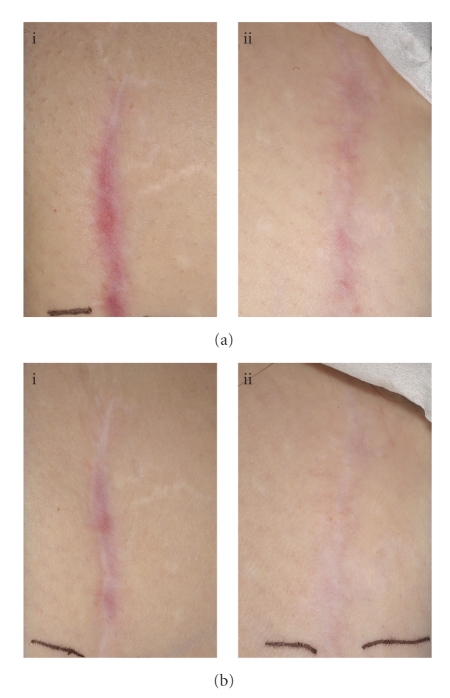
Photographic images showing the improvements in scarring following scar revision surgery with avotermin versus placebo at Month 7 (a) and Month 12 (b). Sections of mature linear scars were randomised to receive placebo (i) or avotermin 200 ng/100 *μ*L (ii) per linear cm immediately following wound closure and 24 hours later.

Although the majority of the phase II studies investigating the safety and efficacy of avotermin have been conducted in volunteers, two recent studies performed in patients demonstrate that the improvements in scarring translate to a clinical setting. Two double-blind, placebo-controlled, randomised, phase II trials in patients demonstrated the scar-improving efficacy and safety of intradermal avotermin administered at a dose of 500 ng/100 *μ*L per linear cm of wound margin on a single occasion following bilateral varicose vein surgery, or administered at a dose of 200 ng/100 *μ*L per linear cm of wound margin on two occasions (immediately after wound closure and 24 hours later) in patients undergoing scar revision surgery (Figure 3). The mechanism of action associated with the macroscopic benefits in scarring reported by assessment panels, investigators, and the patients undergoing scar revision surgery in this study was confirmed by histological improvements in the architecture of the scars, with improved epidermal restitution and an organisation of the extracellular matrix of the papillary and reticular dermis which more closely resembles normal skin. 

Key learnings from the extensive phase I/II clinical trial programme have been used to optimise the design of a phase III study to further evaluate the potential benefits of administering avotermin to improve scar appearance (ClinicalTrial.gov NCT00742443). This study is a randomised double-blind, within-patient, placebo-controlled trial to investigate the efficacy of avotermin in conjunction with scar revision surgery for the improvement of disfiguring scars. The effects of avotermin, administered immediately after wounding and 24 hours later, on subsequent scar formation will be assessed by an independent clinical scar assessment panel. The panel will assess digital photographs of scars at 12 months after surgery using the GSCS. The trial is currently under way in patients with disfiguring linear scars that are suitable for revision and data are awaited with interest. 

## 4. Conclusions and Perspectives

As many patients suffer physical and psychological trauma as a result of scarring, there is a medical need for a prophylactic therapy given at the time of surgery to improve the appearance of scars. Current treatments for scarring have limited efficacy and are not supported by data from prospective, robust clinical trials. Consequently there is no well-established standard of care and the management of scarring is often inadequate. While a number of biological agents designed to manipulate the scarring process are in development, avotermin is the most advanced in phase III clinical trials for the improvement of scar appearance following scar revision surgery. An extensive preclinical and clinical programme has shown that avotermin promotes the regeneration of normal skin and improves scar appearance. The ongoing phase III programme is designed to show that avotermin supplements good surgical technique, resulting in less noticeable scars that more closely resemble the surrounding skin following scar revision surgery.

 Avotermin is the first in a new class of prophylactic therapeutics in development for the improvement of scarring and could have a significant impact on the outcome of scarring for patients in the future. 

## Figures and Tables

**Table 1:** Commonly used approaches to manage scarring post surgery.

Approaches currently used to manage scarring post-surgery	
Non-invasive	Invasive
Silicone gel sheeting	Steroid injections
Pressure garments	Lasers
Hydrating creams/ointments	Dermabrasion
	Scar-revision surgery

**Table 2:** Agents in development for the reduction of dermal scarring.

Agents in *preclinical* development for the reduction of dermal scarring		
Company	Agent	Status

First String	polypeptide *α*-connexin	Preclinical
Phylogica	PYC-35B	Preclinical
Sirnaomics	STP-705	Preclinical

Agents in *clinical* development for the reduction of dermal scarring		

Capstone Therapeutics	AZX-100: 24 amino acid peptide analogue of heat shock protein 20, an intracellular actin-relaxing molecule	Phase II
CoDa Therapeutics	Nexagon: an anti-connexin oligonucleotide, shown to increase rate of wound healing	Phase I
Excaliard	antisense inhibitors of Smads, Connective Tissue Growth Factor	Phase II
Renovo	Ilodecakin (Prevascar): recombinant human interleukin 10	Phase II
	Avotermin (Juvista): recombinant human TGF*β*3	Phase III
